# Trends in Use of Robotic Surgery for Privately Insured Patients and Medicare Fee-for-Service Beneficiaries

**DOI:** 10.1001/jamanetworkopen.2023.15052

**Published:** 2023-05-24

**Authors:** Sidra N. Bonner, Jyothi R. Thumma, Justin B. Dimick, Kyle H. Sheetz

**Affiliations:** 1 Department of Surgery, University of Michigan, Ann Arbor; 2 Center for Health Outcomes and Policy, University of Michigan, Ann Arbor; 3 National Clinician Scholars Program, University of Michigan, Ann Arbor; 4 Department of Surgery, Division of Transplant Surgery, University of California, San Francisco

## Abstract

This cohort study evaluates trends in the adoption of robotic surgery among Medicare beneficiaries and privately insured patients for common general surgical procedures.

## Introduction

The extent to which the adoption of robotic surgery varies among patients with different insurance types is unknown. On the one hand, given that payments from private payers are, on average, 224% of Medicare payments, this difference in reimbursement may be a key factor associated with greater adoption of robotic surgery among privately insured patients.^1^ On the other hand, private and public payers may both balance concerns for higher costs by incentivizing the use of robotic surgery where it adds the most value (eg, transitioning from traditional open surgery to a minimally invasive approach). In this cohort study, we evaluated trends in adoption of robotic surgery among Medicare beneficiaries and privately insured patients for common general surgical procedures experiencing the most rapid increase in the use of the robotic platform.

## Methods

We identified 2 cohorts of patients, Medicare Fee-for-Service beneficiaries and a group of privately insured patients in the MarketScan database, undergoing ventral hernia repair, inguinal hernia repair, colectomy, or proctectomy from January 1, 2010, through December 30, 2018. *International Classification of Diseases, Ninth Revision* or *International Statistical Classification of Diseases, Tenth Revision, Clinical Modification *codes were used to identify procedures (eAppendix in Supplement 1). The University of Michigan institutional review board exempted the study and waived informed consent because this was a secondary analysis and data were deidentified, in accordance with 45 CFR §46. We followed the Strengthening the Reporting of Observational Studies in Epidemiology (STROBE) reporting guidelines for cohort studies.

We used multivariate logistic regression to adjust longitudinal trends in the use of robotic, laparoscopic, and open approaches, accounting for patient age, sex, and Elixhauser comorbidities.^2^ Risk-adjusted proportions are shown throughout. Data were analyzed from July to August 2022 using STATA/MP statistical software version 17 (StataCorp).

## Results

A total of 1 668 697 Medicare beneficiaries and 616 129 privately insured patients underwent the 4 operations studied. The use of robotic surgery for all operations increased from 0.5% in 2010 to 11.9% in 2018 among Medicare patients (22.3%-fold increase; change per year, 1.6%; 95% CI, 1.6%-1.6%) and from 0.3% to 9.2% among privately insured patients (29.6%-fold increase; change per year, 1.0%; 95% CI, 1.0%-1.0%) (Table). Overall, there was a decline in open surgery among Medicare beneficiaries and privately insured patients (−1.3% per year vs −2.4% per year) in the study period. Notably, for ventral hernia repair and colectomy, the trends in increased robotic adoption among both Medicare beneficiaries and privately insured patients was associated with a decrease in laparoscopic surgery (Figure). Colectomy had the largest increase in robotic use among Medicare patients, from 0.4% to 11.5% (29.1%-fold increase). In contrast, among privately insured patients, inguinal hernia repair had the largest increase in robotic utilization, from 0.04% to 5.5% (154.5%-fold increase).

**Table.** Trends in the Use of Robotic, Laparoscopic, and Open Surgery Among Medicare Beneficiaries and Privately Insured Patients by Specific Procedures, 2010-2018

Procedure	Medicare beneficiaries (n = 1 668 697)^a^				Privately insured patients (n = 616 129)^b^			
	Operations per year, risk-adjusted %		Annual slope, % (95% CI)^c^	Fold difference^d^	Operations per year, risk-adjusted %		Annual slope, % (95% CI)^c^	Fold difference^d^
	2010	2018			2010	2018		
Robotic								
All	0.5	11.9	1.6 (1.6 to 1.6)	22.3	0.3	9.2	1.0 (1.0 to 1.0)	29.6
Inguinal hernia repair	0.3	1.3	0.2 (0.1 to 0.2)	3.9	0.04	5.5	0.6 (0.6 to 0.7)	154.5
Ventral hernia repair	0.5	13.8	1.7 (1.6 to 1.7)	25.9	0.1	8.7	0.9 (0.9 to 1.0)	90.0
Colectomy	0.4	11.5	1.6 (1.6 to 1.6)	29.1	0.6	14.9	1.6 (1.5 to 1.6)	24.8
Proctectomy	1.6	20.7	2.5 (2.4 to 2.5)	13.2	2.3	22.7	2.3 (2.1 to 2.4)	10.0
Laparoscopic								
All	21.4	22.2	0.03 (0.01 to 0.06)	1.0	27.9	39.6	1.5 (1.4 to 1.5)	1.4
Inguinal hernia repair	16.1	37.5	2.6 (2.5 to 2.7)	2.3	28.0	49.3	2.6 (2.5 to 2.7)	1.8
Ventral hernia repair	25.8	15.9	−1.3 (−1.4 to −1.2)	0.6	19.0	15.5	−0.5 (−0.6 to −0.3)	0.8
Colectomy	24.1	22.3	−0.4 (−0.4 to −0.3)	0.9	33.8	30.8	−0.4 (−0.5 to −0.3)	0.9
Proctectomy	3.9	14.8	1.9 (1.9 to 2.0)	3.8	4.6	14.1	1.5 (1.3 to 1.6)	3.1
Open								
All	78.2	65.9	−1.5 (−1.5 to −1.4)	0.8	71.8	51.0	−2.5 (−2.5 to −2.4)	0.7
Inguinal hernia repair	83.5	61.1	−2.7 (−2.8 to −2.6)	0.7	71.9	45.1	−3.2 (−3.2 to −3.1)	0.6
Ventral hernia repair	73.7	70.2	−0.3 (−0.4 to −0.2)	1.0	80.9	75.9	−0.5 (−0.6 to −0.3)	0.9
Colectomy	75.7	66.2	−1.0 (−1.0 to −0.98)	0.9	65.6	54.3	−1.3 (−1.4 to −1.2)	0.8
Proctectomy	94.7	64.6	−4.3 (−4.3 to −4.2)	0.7	93.1	63.2	−3.7 (−3.8 to −3.5)	0.7

^a^ For Medicare beneficiaries undergoing surgery between 2010 and 2018, data were collected from MEDPAR files.

^b^ For privately insured patients undergoing surgery between 2010 and 2018, data were collected from MarketScan.

^c^ Refers to annual increase or decrease in the proportional use of each approach by operation.

^d^ Fold difference was defined by dividing the proportional use of a given approach in 2018 by the proportional use in 2010.

**Figure.**
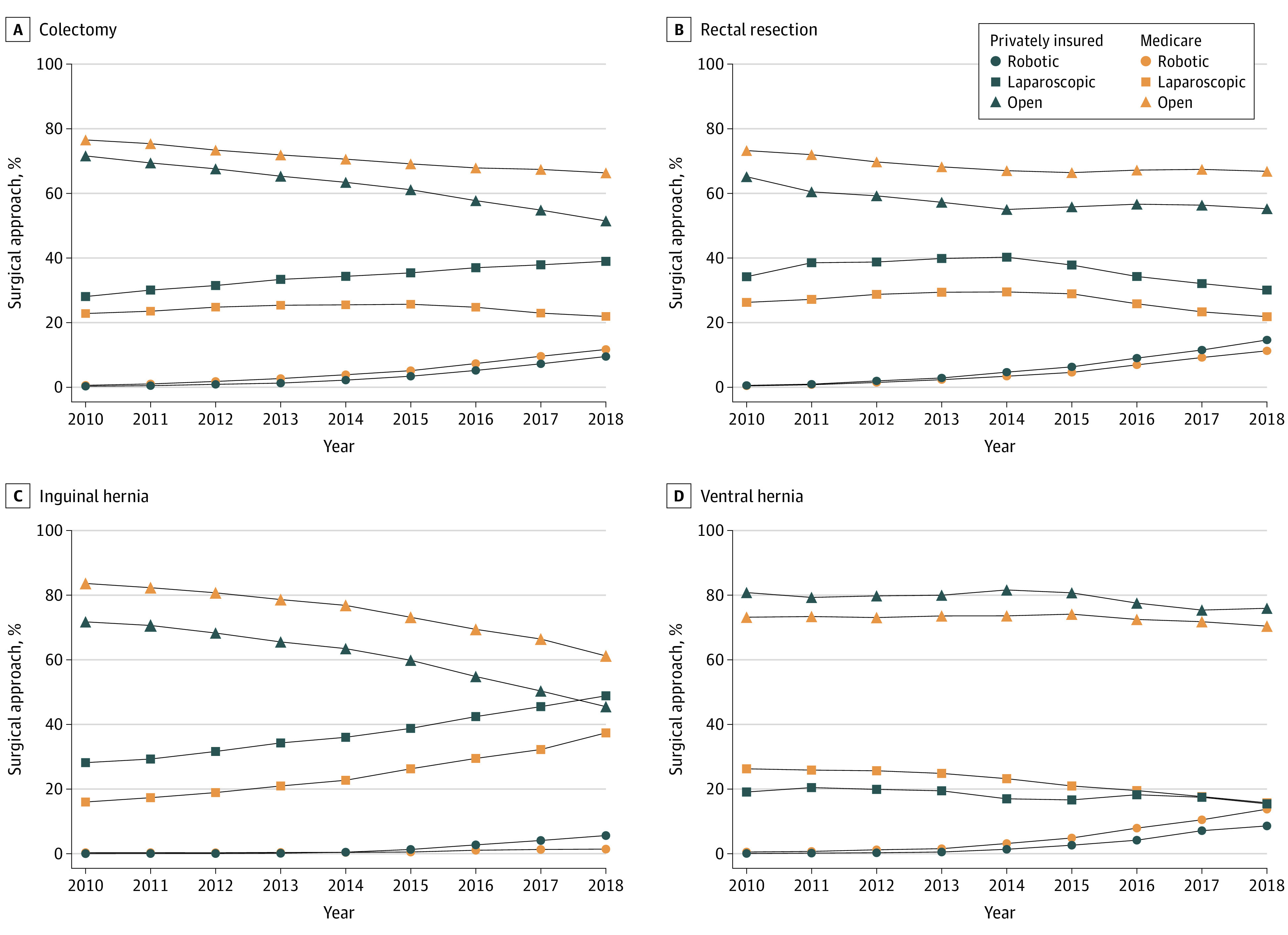
Temporal Trends in the Proportional Use of Robotic, Laparoscopic, and Open Surgery Among Privately Insured Patients vs Medicare Beneficiaries Graphs show data for surgical trends in colectomy (A), rectal resection (B), inguinal hernia (C), and ventral hernia (D) from 2010 to 2018.

## Discussion

The findings of this cohort study demonstrate that the adoption of robotic surgery for common general surgical procedures was similar between Medicare Fee-for-Service beneficiaries and privately insured patients. This suggests that potential differences in reimbursement and potential differences in consumer preferences between younger patients with employee-sponsored insurance compared with older adults with Medicare do not appear to be associated with adoption. However, for both cohorts, the magnitude of increase in robotic surgery adoption appears to outpace the relative increase in laparoscopic approaches and decrease in open approaches. Similar to prior findings,^3^ we found evidence for the replacement of both open and laparoscopic techniques with robotic surgery for common operations. The replacement of laparoscopic surgery with robotic approaches requires ongoing evaluation given the unclear clinical benefits and increase costs and resource allocation.^4,5^

Our results should be interpreted within the context of several limitations. This study is limited by changes in patient and surgeon factors that may contribute to surgical approach, such as complexity of hernia repair or increased training in robotic surgery by surgical trainees and practicing surgeons.^5^ However, given the lack of consensus regarding patient selection for robotic approaches and variation in surgeon robotic training, inclusion of these factors could introduce potential bias.^6^ Furthermore, our study does not address how nonclinical factors, such as advertisement of robotic surgery, may contribute to patient preference for robotic approaches regardless of payer. Research centered on the evaluation adoption of robotic surgery will require ongoing considerations of hospital, surgeon, and patient factors.

## Notes

Supplement 1. **eAppendix.** Codes Used for Operations

Supplement 2.
Data Sharing Statement
