# Supplementary material for: Trends in Use of Robotic Surgery for Privately Insured Patients and Medicare Fee-for-Service Beneficiaries
Source: JAMA Netw Open. 2023 May 24;6(5):e2315052. doi: 10.1001/jamanetworkopen.2023.15052 (PMC10209745; doi:10.1001/jamanetworkopen.2023.15052)
Supplement: Supplement 1. — eAppendix. Codes Used for Operations [file jamanetwopen-e2315052-s001.pdf]

Supplemental Online Content

Bonner SN, Thumma JR, Dimick JB, Sheetz KH. Trends in use of robotic surgery for privately insured patients and Medicare fee-for-service beneficiaries. *JAMA Netw Open*. 2023;6(5):e2315052. doi:10.1001/jamanetworkopen.2023.15052

eAppendix. Codes Used for Operations

This supplemental material has been provided by the authors to give readers additional information about their work.

© 2023 Bonner SN et al. *JAMA Network Open.*

eAppendix. Codes Used for Operations

Robotic -
'S2900','1741','1742','1743','1744','1745','17416','1747','1748','1749','8E0W0CZ','8E0W3CZ','8E0W4CZ','8E0W7CZ',' 8E0W8CZ'

Colectomy:

Lap colectomy -1731- 1739, 0DTH4ZZ, 0DTE4ZZ, 0DTF4ZZ, 0DTL4ZZ, 0DTG4ZZ, 0DTN4ZZ, 0DBE4ZZ,'0DBF4ZZ, 0DBG4ZZ, 0DBH4ZZ, 0DBK4ZZ, 0DBL4ZZ, 0DBM4ZZ, 0DBN4ZZ, 0DTK4ZZ, 0DTM4ZZ

Open - 457, 4571-4576, 4579, 458, 4581-4583, 0DBE0ZZ, 0DBE3ZZ, 0DBE7ZZ, 0DBF0ZZ, 0DBF3ZZ, 0DBF7ZZ, 0DBG0ZZ, 0DBG3ZZ, 0DBG7ZZ, 0DBH0ZZ, 0DBH3ZZ, 0DBH7ZZ, 0DBK0ZZ, 0DBK3ZZ, 0DBK7ZZ, 0DBL0ZZ, 0DBL3ZZ, 0DBL7ZZ, 0DBM0ZZ, 0DBM3ZZ, 0DBM7ZZ, 0DBN0ZZ, 0DBN3ZZ, 0DBN7ZZ, 0DTE0ZZ, 0DTE7ZZ, 0DTF0ZZ, 0DTF7ZZ, 0DTG0ZZ, 0DTG7ZZ, 0DTH0ZZ, 0DTH7ZZ, 0DTK0ZZ, 0DTK7ZZ, 0DTL0ZZ, 0DTL7ZZ, 0DTM0ZZ, 0DTM7ZZ, 0DTN0ZZ, 0DTN7ZZ

Rectal resection:
Open - 4840, 4841, 4843-4849, 4850, 4852-4859, 4860-4869, 0DBP0ZZ, 0DTP0ZZ, 0DTP7ZZ

Lap – 4842, 4851, 0DTP4ZZ, 0D1N0Z4

Ventral/incisional hernia:
Open – 535, 5351, 5359, 536, 5361, 5363, 5369, 0WQF0ZZ, 0WMF0ZZ, 0WQF0ZZ, 0WUF07Z, 0WUF0JZ, 0WUF0KZ, 0WQFXZZ

Lap – 5362, 0WQF3ZZ, 0WQF4ZZ, 0WUF47Z, 0WUF4JZ, 0WUF4KZ

Inguinal hernia –

open – 5300, 5301, 5302, 5303, 5304, 5305, 5310, 5311, 5312, 5313, 5314, 5315, 5316, 5317, 0YQ50ZZ, 0YQ60ZZ, 0YQA0ZZ, 0YU507Z, 0YU50JZ, 0YU50KZ, 0YU607Z, 0YU60JZ, 0YU60KZ, 0YUA07Z, 0YUA0JZ, 0YUA0KZ

lap – 1711, 1712, 1713, 1721, 1722, 1723, 1724, 0YQ53ZZ, 0YQ54ZZ, 0YQ63ZZ, 0YQ64ZZ, 0YQA3ZZ, 0YQA4ZZ, 0YU547Z, 0YU54JZ, 0YU54KZ, 0YU647Z, 0YU64JZ, 0YU64KZ, 0YUA47Z, 0YUA4JZ, 0YUA4KZ

© 2023 Bonner SN et al. *JAMA Network Open.*
