# Supplementary material for: Trends in Use of Robotic Surgery for Privately Insured Patients and Medicare Fee-for-Service Beneficiaries
Source: JAMA Netw Open. 2023 May 24;6(5):e2315052. doi: 10.1001/jamanetworkopen.2023.15052 (PMC10209745; doi:10.1001/jamanetworkopen.2023.15052)
Supplement: Supplement 2. — Data Sharing Statement [file jamanetwopen-e2315052-s002.pdf]

Data Sharing Statement

Bonner. Trends in Use of Robotic Surgery for Privately Insured Patients and Medicare Fee-For-Service Beneficiaries. *JAMA Netw Open*. Published May 24, 2023. doi:10.1001/jamanetworkopen.2023.15052

Data
Data available: No

Additional Information
Explanation for why data not available: Medicare Data is publicly available and de-identified.
